# PinSnps: structural and functional analysis of SNPs in the context of protein interaction networks

**DOI:** 10.1093/bioinformatics/btw153

**Published:** 2016-03-24

**Authors:** Hui-Chun Lu, Julián Herrera Braga, Franca Fraternali

**Affiliations:** Randall Division of Cell and Molecular Biophysics, King’s College London, London SE1 1UL, UK

## Abstract

**Summary:** We present a practical computational pipeline to readily perform data analyses of protein–protein interaction networks by using genetic and functional information mapped onto protein structures. We provide a 3D representation of the available protein structure and its regions (surface, interface, core and disordered) for the selected genetic variants and/or SNPs, and a prediction of the mutants’ impact on the protein as measured by a range of methods. We have mapped in total 2587 genetic disorder-related SNPs from OMIM, 587 873 cancer-related variants from COSMIC, and 1 484 045 SNPs from dbSNP. All result data can be downloaded by the user together with an R-script to compute the enrichment of SNPs/variants in selected structural regions.

**Availability and Implementation:** PinSnps is available as open-access service at http://fraternalilab.kcl.ac.uk/PinSnps/

**Contact:** franca.fraternali@kcl.ac.uk

**Supplementary information:** Supplementary data are available at *Bioinformatics* online.

## 1 Introduction

High-throughput experiments are routinely performed to decipher genetic, metabolic and protein-protein interaction networks (PPINs) and bioinformaticians are compelled to develop efficient and accurate tools to assist decision-making based on available data from multiple sources (Chung *et al.*, 2015; Fernandes *et al.*, 2010; Lu *et al.*, 2013). Bioinformatics applications, which merge available genomic, interaction and structural data, can be broadly classified into exploratory or predictive tools. The former comprises of tools which map and visualize the merged data (Kelley *et al.*, 2015; Lees *et al.*, 2010; Mosca *et al.*, 2015; Niknafs *et al.*, 2013; Pappalardo and Wass, 2014; Ryan *et al.*, 2009; Vazquez *et al.*, 2015), while predictive tools are quantitative estimators of the potential impact of SNPs/variants and offer an assessment in terms of scores or pseudo free-energy metrics (Adzhubei *et al.*, 2010; Betts *et al.*, 2015; Li *et al.*, 2014; Ng and Henikoff, 2003; Pires *et al.*, 2014a; Pires *et al.*, 2014b; Pires *et al.*, 2016; Yates *et al.*, 2014).

**Fig. 1.**
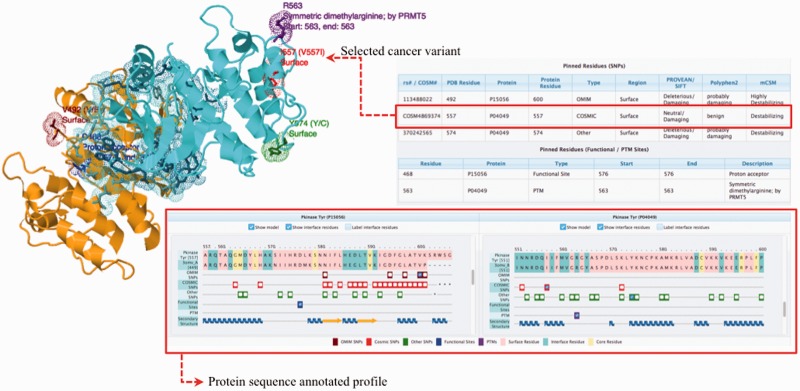
PinSnps user interface overview. The complex between Raf1 (P04049, coloured in cyan) and Braf (P15056, coloured in orange) is shown. The protein sequence annotated profile of the complex shows the sequence alignment of the query protein sequence and the available PDB structure sequences. A more detailed description of the platform interactive output is given in the Supplementary Figure S1

In this application, we use 3D interactome networks and their homologs to highlight how human variants and disease-causing mutations may affect protein function and complex stability. Recent studies have used the structural information of PPINs to understand the molecular mechanisms of binding partner selection (Fornili *et al.*, 2013). These reliable methods only consider the interactions that have a representative 3D structure or a close homolog with a 3D structure to add weight to the existence of the observed protein interactions (or network links) in a given PPIN (Hooda and Kim, 2012; Kim *et al.*, 2006; Lees *et al.*, 2011; Meyer *et al.*, 2013; Mosca *et al.*, 2013; Wang *et al.*, 2012). Multiple studies have pointed out that the interfaces of protein complexes harbours mutations associated with diseases (Espinosa *et al.*, 2014; Gao *et al.*, 2015; Kamburov *et al.*, 2015; Nishi *et al.*, 2013; Studer *et al.*, 2013; Wang *et al.*, 2012; Yates and Sternberg, 2013a,b). The evaluation of the impact of genomic variation on coding regions can be enhanced by mapping SNPs to distinct regions of protein structure, i.e. surface, interface or core. To generate a comprehensive mapping of available SNPs onto PPINs, the automatic pipeline PinSnps has been developed (for details see Supplementary Fig. S2); this extracts structure-integrated human PPINs, enriched with information from homologous protein domains with sequence identity higher than 30%. The main strengths and differences to previous approaches lie in (i) the use of homologous structures of human protein sequences in the PPINs to map the studied variants, which more than doubles the available positional 3D information; (ii) the mapping onto predefined protein regions (surface, core, interface) along with the mapping of functional sites and Post-Translational Modifications (PTMs) (obtained from UniProt (UniProt Consortium, 2015)). This information, together with precompiled predictions of the SNP/variant’s impact from multiple predictors, can help users to quantitatively assess and evaluate the functional implications of their studied variants. The annotation of both intra- and inter-domain disordered regions as predicted by DISOPRED2 (Ward *et al.*, 2004) has also been included in the pipeline, as recent studies imply the importance of these regions in regulating biological functions (Cline and Karchin, 2011; Gibbs and Showalter, 2015; Wright and Dyson, 2015); (iii) allowing the users to download the query data in various file formats (Fig. 1).

## 2 Implementation and features

The PPIN used in this study has been derived as a non-redundant set of protein interactions from the list of human PPIs given in Supplementary Table S1. The current release includes data of 16 603 proteins, of which 4673 have a resolved structure and 4962 have a homologous structure (Supplementary Fig. S3).

PinSnps is, to our knowledge, one of the largest collections of variants mapped onto 3D coordinates. SNPs from dbSNP (Sherry *et al.*, 2001), consisting of common and germ-line disease variants (the later originally from OMIM (Hamosh *et al.*, 2005)), together with somatic cancer mutations from COSMIC (Forbes *et al.*, 2015) have been mapped onto cognate 3D structures and, when not available, to their homologous structures. The use of homologous structures expands significantly the number of SNPs/variants mapped onto 3D positions within folded domains. The enrichment of disease-associated variants in specific regions of proteins can be quantified using Formula S1 and the R script which is provided on the PinSnps ‘Downloads’ webpage (see example in Supplementary Fig. S4).

We present a number of case studies and more detailed instructions on the web server’s ‘Help’ page and in the Supplementary Materials.

### 2.1 Protein sequence annotated profiles

Each protein in the PPIN is transformed into a sequence-annotated string (we refer to this as ‘profile’) that represents the fingerprint of the user-selected information. These profiles were generated based on information obtained from sequence alignments, available structural information, human genetic data (from dbSNP, OMIM and COSMIC) and UniProt protein functional site and PTM annotations. PSI-BLAST (Altschul *et al.*, 1997) was used to identify resolved and homologous structures of human proteins by searching against sequences of the Protein Data Bank (Berman *et al.*, 2000). Homologous structures with more than 80% coverage of the human protein domain sequence and with more than 30% sequence identity were selected. Each protein was annotated with domain boundaries according to Pfam (Finn *et al.*, 2014). Alignments between sequences of query protein domains and available protein structure sequences were performed using T-Coffee (Notredame *et al.*, 2000). The classification of structural regions, i.e. the definition of surface, interface and core regions, was based on the surface area analysis of POPSCOMP (Kleinjung and Fraternali, 2005).

*Conflict of Interest*: none declared.

## Supplementary Material

Supplementary Data
