# Supplementary material for: PinSnps: structural and functional analysis of SNPs in the context of protein interaction networks
Source: Bioinformatics. 2016 Mar 24;32(16):2534–6. doi: 10.1093/bioinformatics/btw153 (PMC4978923; doi:10.1093/bioinformatics/btw153)
Supplement: Supplementary Data [file supp_32_16_2534__index.html]

PinSnps: structural and functional analysis of SNPs in the context of protein interaction networks — PinSnps: structural and functional analysis of SNPs in the context of protein interaction networks — Supplementary Data 

# PinSnps: structural and functional analysis of SNPs in the context of protein interaction networks

## Supplementary Data

files

- Supplementary Data - docx file
