# Supplementary material for: PinSnps: structural and functional analysis of SNPs in the context of protein interaction networks
Source: Bioinformatics. 2016 Mar 24;32(16):2534–6. doi: 10.1093/bioinformatics/btw153 (PMC4978923; doi:10.1093/bioinformatics/btw153)
Supplement: Supplementary Data [file supp_btw153_bioinformatics_application_note_PinSnps_supplementary_online.docx]

Supplementary Materials

Web server implementation

PinSnps is a Java web application whose core architecture uses Java EE. The web services are hosted on an Apache Tomcat webserver. JSmol (<http://jsmol.sourceforge.net>) is used to visualise protein complexes and Cytoscape (<http://www.cytoscape.org>) is used to display Protein-Protein Interaction Networks. The mapping of human genetic data onto protein structures in the human PPIN was precompiled and stored as text files that are read and displayed by the web application.

Web server use

We will describe three of the most common use-cases of the PinSnps server as ‘Case study 1: Protein Network mining to search for specific interactions, ‘Case study 2: Application of PinSnps data’ and ‘Case Study 3: Protein Information’. We focus on the relationship between the interactive menu and the underlying data sources for the query proteins and describe an application of the downloadable data analysis with an R-script available from the server.

Input

Input can be (i) a protein identifier, provided as UniProt accession number to search for a single protein or a sub-network of interactors; (ii) dbSNP entries to query specific SNPs; (iii) COSMIC entries to search whithin a cancer tissue type.

Output

The interactive user-interface comprises: 3D view of a single protein or protein complexes; sequence alignments (query sequence and 3D structure sequence) showing SNPs, PTMs and functional sites as checkboxes selectable by the user and labeled on the interactive rotatable 3D structure.

The returned pages ‘Protein Information’ (single protein query) or ‘Protein-Protein Interactions’ (protein network query) are partitioned into a vertically organised layout with expandable panels containing information from different data sources. All panels can be unfolded by selecting the ‘+’ symbol on the panel bar or folded by selecting the ‘-’ symbol. Only the domain panel is expanded by default to provide an initial overview of the query protein domains.

One of the important features of PinSnps is that it provides pre-calculated SNPs Impact Predictions obtained from the methods: Provean/Sift, Polyphen2 and mCSM (protein stability assessment only). We are, to our knowledge, the first to introduce pre-calculated values from mCSM into a large-scale data analysis pipeline. We report only the protein stability impact of single nucleotide change on the structure using this method. When the prediction is reported as N.A. on the PinSnps webpage, this is due to a difference in amino acid type at that specific position between the original query protein and the crystal structure sequence. The number of the SNPs/variants occurring at these positions has been summarised: in the case of **COSMIC,** 2154 out of 237191 have been found to be at positions with a different a.a. in the selected solved structures; 7742 out of 753736 SNPs for **dbSNP** and 70 out of 5929 SNPs for **OMIM**.

Case study 1: Protein network mining for specific interactions

The serine/threonine protein kinase Raf1 (UniProt AC P04049) belongs to the Ras subfamily of proteins. Members of this family act as regulatory hubs by linking to a diverse set of cellular signalling pathways. The protein interaction network of Raf1 can be explored by entering its UniProt accession number P04046 into the ‘Protein Network’ entry field on the PinSnps front page. By selecting ‘Query’, PinSnps will launch the data retrieval and return a table of binary interactions.

To obtain the results shown in Figure S1, the complex between Raf1 and Braf (P15056) is selected by clicking on the button in the ‘Structure’ column of the ‘Interactions’ table. The complex is shown in the structural view panel (JSmol); the interface region of the binary complex can be shown by selecting ‘Show Interface Residues’ (rendered in cyan in Figure S1). SNPs/variants, functional sites, and PTMs can also be explored by selecting the colour-coded boxes in the sequence profile panel. The selected residues will then be annotated on the complex and listed in the ‘Pinned Residues’ tables. In the example (Figure S1), the residues V600 ‘OMIM SNPs’ and D576 ‘Functional Sites’ from Braf, and I557 ‘COSMIC SNPs’, Y574 ‘Other SNPs’ and R563 ‘PTM’ from Raf1 have been selected.

PinSnps also provides a search function for a single protein structure and its SNPs/variants information by either entering a UniProt accession number into the ‘Single Protein’ entry field on the front page or selecting the protein accession number or the Gene Symbol in the ‘Interactions’ table retrieved from ‘Protein Network’ (see Case Study 3 for an example).

By selecting the ‘View Network’ button or by expanding the ‘Network of Structure’ panel on the ‘Protein-Protein Interactions’ page, a colour-coded network is displayed, where the query protein(s) is shown as green triangle and the interactors that have a known 3D complex structure (human or homologous species) are shown as red circles.

Case Study 2: Application of PinSnps data

In Figure S3, an application of the result data from PinSnps is shown. By using the enrichment formula implemented in the downloadable R-script (on the ‘Downloads’ page) we have calculated the occurrence of SNPs/variants close to PTMs and functional sites. We used a per-residue 3D search with a cut-off of 8 Å. Protein domains were divided into two categories, kinase and non-kinase domains. Protein kinase domains contain the catalytic function as annotated by Pfam (Finn, et al., 2014) and are involved in the process of phosphorylation. Our results show that SNPs/variants within kinase domains are enriched in the vicinity of PTMs or functional sites when compared to the non-kinase domains. COSMIC variants are also significantly enriched (as calculated by Equation S1); the statistical significance was estimated with a Student’s t-test on the confidence intervals (Cumming, et al., 2007; Lu, et al., 2015; Wolfe and Hanley, 2002)) in the regions close to PTMs and functional sites, as expected considering the crucial role of kinases in cancer. Similar results are observed for the OMIM germ-line variants.

Case study 3: Protein Information

The receptor-type tyrosine-protein kinase FLT3 (P36888) is a key regulator of the development of hematopoietic and immune cells. Mutational activation of FLT3 is a driving force in the development of acute myeloid leukemia. Entering the accession number and pressing ‘Query’ will launch the data retrieval. The returned page ‘Protein Information’ is partitioned into vertically organised layers: a link to the protein’s UniProt page, a bar of shortcut buttons to the following sections and a stack of collapsed panels. Each panel contains information from a different data source, in top-to-bottom order: included ‘Protein Domains’, ‘Germ-line Disease SNPs (OMIM)’, ‘Somatic Cancer SNVs (COSMIC)’, ‘Other Known SNPs’, ‘Functional Sites’ and ‘Post-Translational Modifications (PTMs)’. Selecting the arrow symbol in the ‘View Structures’ column for the ‘Pkinase_Tyr’ domain reveals all available domain structures, including homologs with sequence identity over 30% together with relevant alignment data. The arrow symbol for the entry ‘PDB ID’ 3u6j_A in the expanded list ‘Structure Models’ opens a new panel containing a rotatable structure window and on the bottom a colour-coded alignment (Figure S6) between the query protein and the PDB structure sequence. Selection of the colour-coded checkboxes in the alignment triggers rendering and annotation of the respective side-chain in the structure viewer and listing in the ‘Pinned Residues’ table. To reproduce Figure S6, select D811 ‘Functional Sites’, G822 ‘COSMIC SNPs’, D835 ‘OMIM SNPs, R845 ‘Other SNPs’ and Y842 ‘PTM’, where the numbers refer to the P36888 UniProt protein sequence.

Most of the panel entries on the ‘Protein Information’ page are self-explanatory; however, it is noteworthy that the entries ‘Region-Disordered’, ‘Provean/SIFT’, ‘PolyPhen2’, and ‘mCSM’ in the ‘Germ-line Disease SNPs’, ‘Somatic Cancer SNVs’ and ‘Other SNPs’ panels are estimates from predictions, in contrast to the deterministic nature of all other displayed data.

Acknowledgements

The authors thank Jens Kleinjung for technical and scientific assistance throughout the project. The authors are also grateful to Sun Sook Chung, Arianna Fornili, Anna Laddach for testing the application.

Funding

This work was supported by BBSRC (BB/G017190/1) and MRC (MR/L01257X/1).

Conflict of Interest: none declared.

References

Adzhubei, I.A., et al. A method and server for predicting damaging missense mutations. Nat Methods 2010;7(4):248-249.

Altschul, S.F., et al. Gapped BLAST and PSI-BLAST: a new generation of protein database search programs. Nucleic Acids Res 1997;25(17):3389-3402.

Berman, H.M., et al. The Protein Data Bank. Nucleic Acids Res 2000;28(1):235-242.

Betts, M.J., et al. Mechismo: predicting the mechanistic impact of mutations and modifications on molecular interactions. Nucleic Acids Res 2015;43(2):e10.

Chung, S.S., et al. Bridging topological and functional information in protein interaction networks by short loops profiling. Sci Rep 2015;5:8540.

Cline, M.S. and Karchin, R. Using bioinformatics to predict the functional impact of SNVs. Bioinformatics 2011;27(4):441-448.

Cumming, G., Fidler, F. and Vaux, D.L. Error bars in experimental biology. J Cell Biol 2007;177(1):7-11.

Espinosa, O., et al. Deriving a mutation index of carcinogenicity using protein structure and protein interfaces. PLoS One 2014;9(1):e84598.

Fernandes, L.P., et al. Protein networks reveal detection bias and species consistency when analysed by information-theoretic methods. PLoS One 2010;5(8):e12083.

Finn, R.D., et al. Pfam: the protein families database. Nucleic Acids Res 2014;42(Database issue):D222-230.

Forbes, S.A., et al. COSMIC: exploring the world's knowledge of somatic mutations in human cancer. Nucleic Acids Res 2015;43(Database issue):D805-811.

Fornili, A., et al. Specialized Dynamical Properties of Promiscuous Residues Revealed by Simulated Conformational Ensembles. J Chem Theory Comput 2013;9(11):5127-5147.

Gao, M., Zhou, H. and Skolnick, J. Insights into Disease-Associated Mutations in the Human Proteome through Protein Structural Analysis. Structure 2015;23(7):1362-1369.

Gibbs, E.B. and Showalter, S.A. Quantitative biophysical characterization of intrinsically disordered proteins. Biochemistry 2015;54(6):1314-1326.

Hamosh, A., et al. Online Mendelian Inheritance in Man (OMIM), a knowledgebase of human genes and genetic disorders. Nucleic Acids Res 2005;33(Database issue):D514-517.

Hooda, Y. and Kim, P.M. Computational structural analysis of protein interactions and networks. Proteomics 2012;12(10):1697-1705.

Kamburov, A., et al. Comprehensive assessment of cancer missense mutation clustering in protein structures. Proc Natl Acad Sci U S A 2015;112(40):E5486-5495.

Kelley, L.A., et al. The Phyre2 web portal for protein modeling, prediction and analysis. Nat Protoc 2015;10(6):845-858.

Kim, P.M., et al. Relating three-dimensional structures to protein networks provides evolutionary insights. Science 2006;314(5807):1938-1941.

Kleinjung, J. and Fraternali, F. POPSCOMP: an automated interaction analysis of biomolecular complexes. Nucleic Acids Res 2005;33(Web Server issue):W342-346.

Lees, J., et al. Gene3D: merging structure and function for a Thousand genomes. Nucleic Acids Res 2010;38(Database issue):D296-300.

Lees, J.G., et al. Systematic computational prediction of protein interaction networks. Phys Biol 2011;8(3):035008.

Li, M., et al. Predicting the Impact of Missense Mutations on Protein-Protein Binding Affinity. J Chem Theory Comput 2014;10(4):1770-1780.

Lu, H.C., et al. Anatomy of protein disorder, flexibility and disease-related mutations. Front Mol Biosci 2015;2:47.

Lu, H.C., Fornili, A. and Fraternali, F. Protein-protein interaction networks studies and importance of 3D structure knowledge. Expert Rev Proteomics 2013;10(6):511-520.

Meyer, M.J., et al. INstruct: a database of high-quality 3D structurally resolved protein interactome networks. Bioinformatics 2013;29(12):1577-1579.

Mosca, R., Ceol, A. and Aloy, P. Interactome3D: adding structural details to protein networks. Nat Methods 2013;10(1):47-53.

Mosca, R., et al. dSysMap: exploring the edgetic role of disease mutations. Nat Methods 2015;12(3):167-168.

Ng, P.C. and Henikoff, S. SIFT: Predicting amino acid changes that affect protein function. Nucleic Acids Res 2003;31(13):3812-3814.

Niknafs, N., et al. MuPIT interactive: webserver for mapping variant positions to annotated, interactive 3D structures. Hum Genet 2013;132(11):1235-1243.

Nishi, H., et al. Cancer missense mutations alter binding properties of proteins and their interaction networks. PLoS One 2013;8(6):e66273.

Notredame, C., Higgins, D.G. and Heringa, J. T-Coffee: A novel method for fast and accurate multiple sequence alignment. J Mol Biol 2000;302(1):205-217.

Pappalardo, M. and Wass, M.N. VarMod: modelling the functional effects of non-synonymous variants. Nucleic Acids Res 2014;42(Web Server issue):W331-336.

Pires, D.E., Ascher, D.B. and Blundell, T.L. DUET: a server for predicting effects of mutations on protein stability using an integrated computational approach. Nucleic Acids Res 2014 A ;42(Web Server issue):W314-319.

Pires, D.E., Ascher, D.B. and Blundell, T.L. mCSM: predicting the effects of mutations in proteins using graph-based signatures. Bioinformatics 2014 B ;30(3):335-342.

Pires, D.E., et al. In silico functional dissection of saturation mutagenesis: Interpreting the relationship between phenotypes and changes in protein stability, interactions and activity. Sci Rep 2016;6:19848.

Ryan, M., et al. LS-SNP/PDB: annotated non-synonymous SNPs mapped to Protein Data Bank structures. Bioinformatics 2009;25(11):1431-1432.

Sherry, S.T., et al. dbSNP: the NCBI database of genetic variation. Nucleic Acids Res 2001;29(1):308-311.

Studer, R.A., Dessailly, B.H. and Orengo, C.A. Residue mutations and their impact on protein structure and function: detecting beneficial and pathogenic changes. Biochem J 2013;449(3):581-594.

UniProt Consortium. UniProt: a hub for protein information. Nucleic Acids Res 2015;43(Database issue):D204-212.

Vazquez, M., Valencia, A. and Pons, T. Structure-PPi: a module for the annotation of cancer-related single-nucleotide variants at protein-protein interfaces. Bioinformatics 2015;31(14):2397-2399.

Wang, X., et al. Three-dimensional reconstruction of protein networks provides insight into human genetic disease. Nat Biotechnol 2012;30(2):159-164.

Ward, J.J., et al. The DISOPRED server for the prediction of protein disorder. Bioinformatics 2004;20(13):2138-2139.

Wolfe, R. and Hanley, J. If we're so different, why do we keep overlapping? When 1 plus 1 doesn't make 2. CMAJ 2002;166(1):65-66.

Wright, P.E. and Dyson, H.J. Intrinsically disordered proteins in cellular signalling and regulation. Nat Rev Mol Cell Biol 2015;16(1):18-29.

Yates, C.M., et al. SuSPect: enhanced prediction of single amino acid variant (SAV) phenotype using network features. J Mol Biol 2014;426(14):2692-2701.

Yates, C.M. and Sternberg, M.J. The effects of non-synonymous single nucleotide polymorphisms (nsSNPs) on protein-protein interactions. J Mol Biol 2013;425(21):3949-3963.

Yates, C.M. and Sternberg, M.J.E. Proteins and domains vary in their tolerance of non-synonymous single nucleotide polymorphisms (nsSNPs). Journal of molecular biology 2013;425(8):1274-1286.


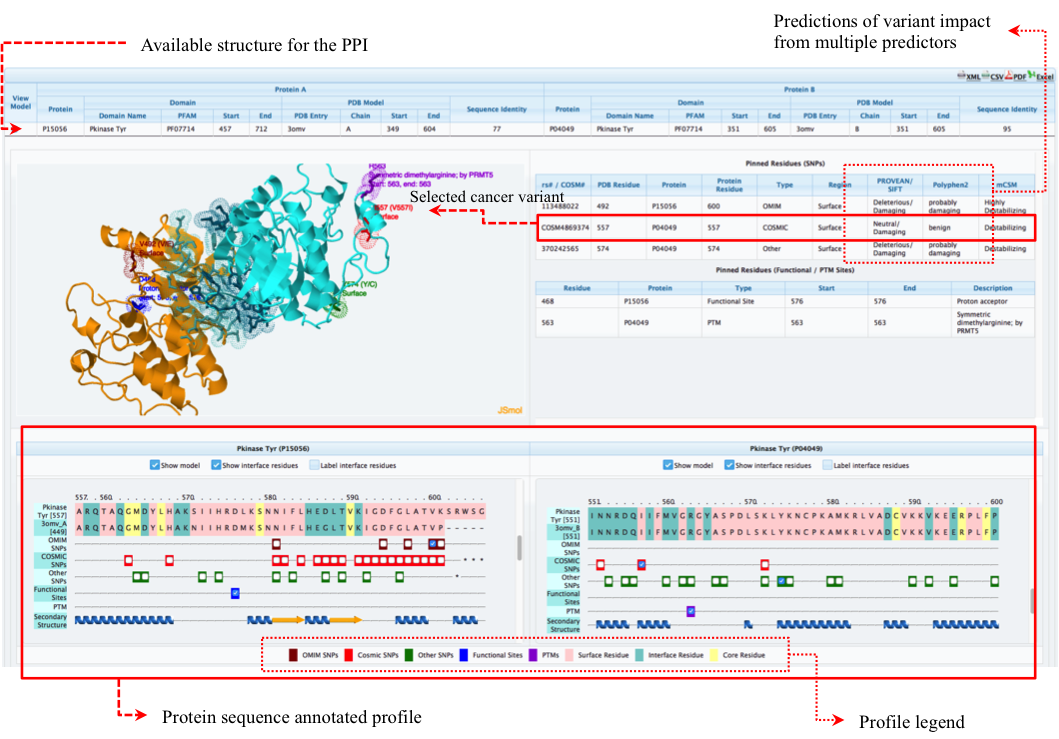
Figure S1. The complex between Raf1 (P04049, coloured in cyan) and Braf (P15056, coloured in orange) is shown. The interface regions of the two protein domains were rendered by selecting ‘Show Interface Residues’. The annotated sequence profile of the protein complex shows the sequence alignments between the protein sequences and the PDB structure sequences coloured according to the protein regions namely surface (pink), interface (green) and core (yellow). The positions of SNPs/variants are shown in separate sequence lanes with the checkboxes allowing the users to select and view them mapped onto the complex structure. Functional sites and PTMs are also annotated in the alignments and can be similarly highlighted in the 3D-structure. The selected residues are listed in tables, where the predictions of the impact of SNPs/variants are given.


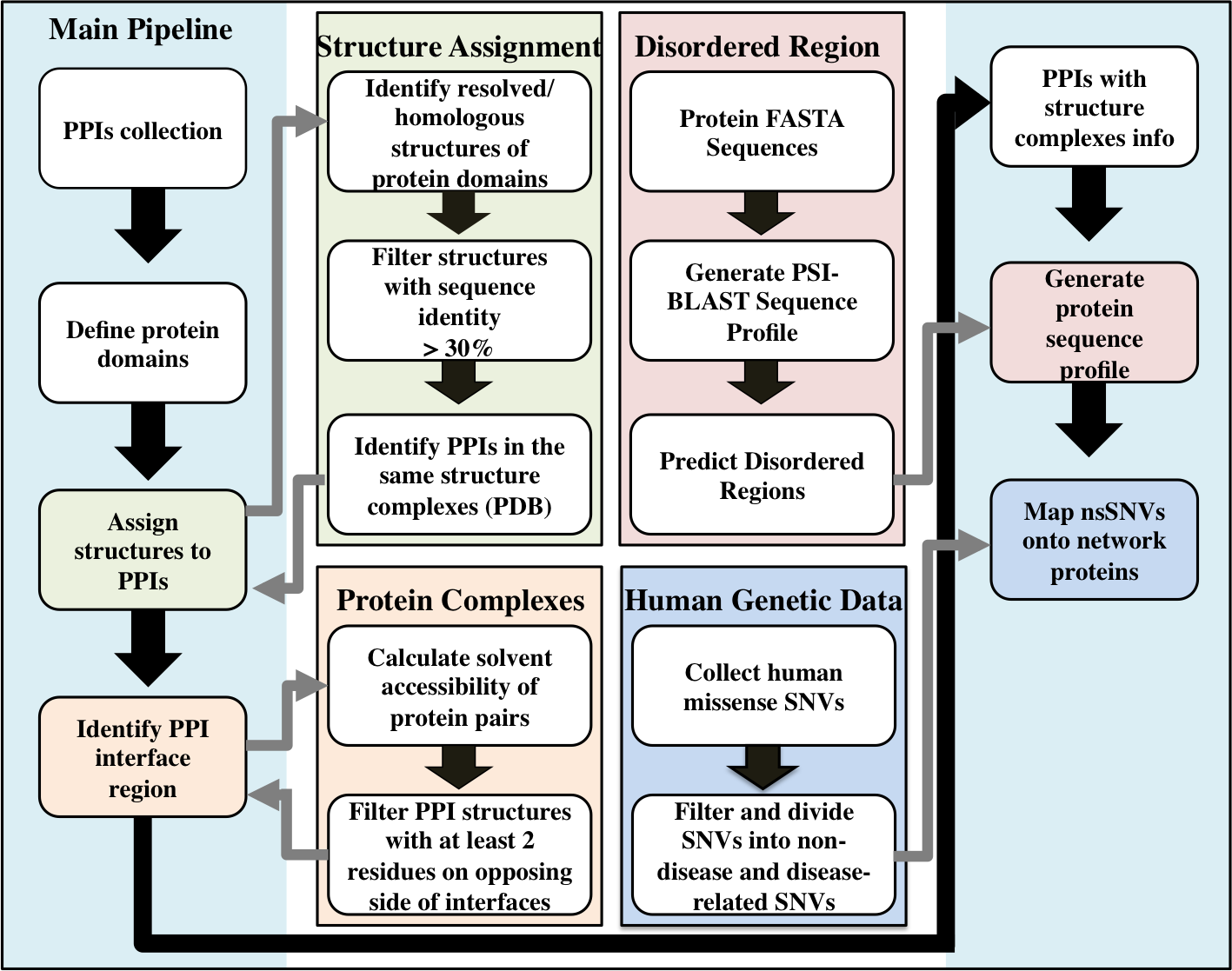


Figure S2. Flowchart of the pipeline used to filter, integrate and map the data. SNVs in the ‘Human Genetic Data’ box stands for Single Nucleotide Variants and is used as a generic term for SNPs and Variants.


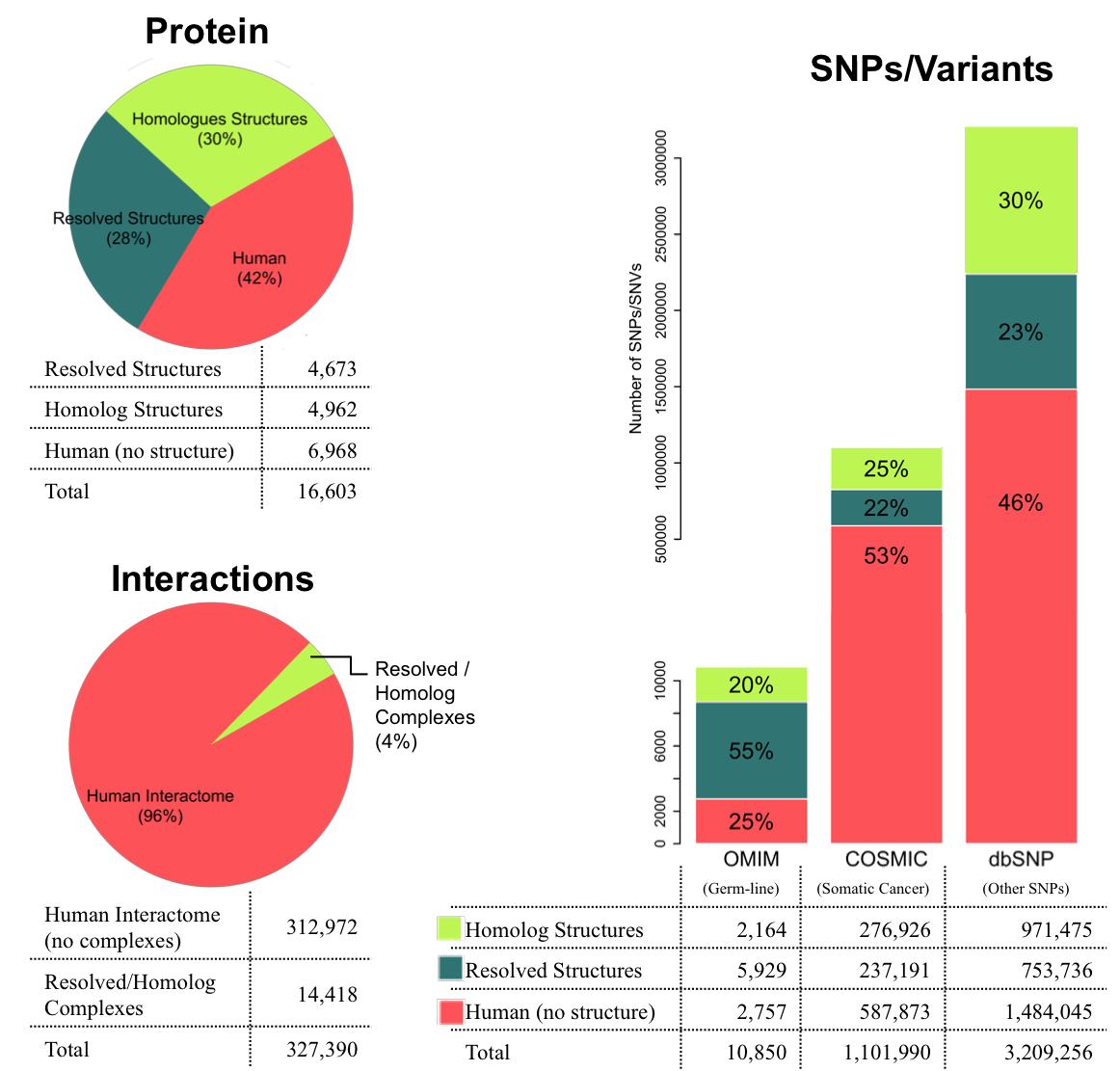


Figure S3. Summary of the mapped data.


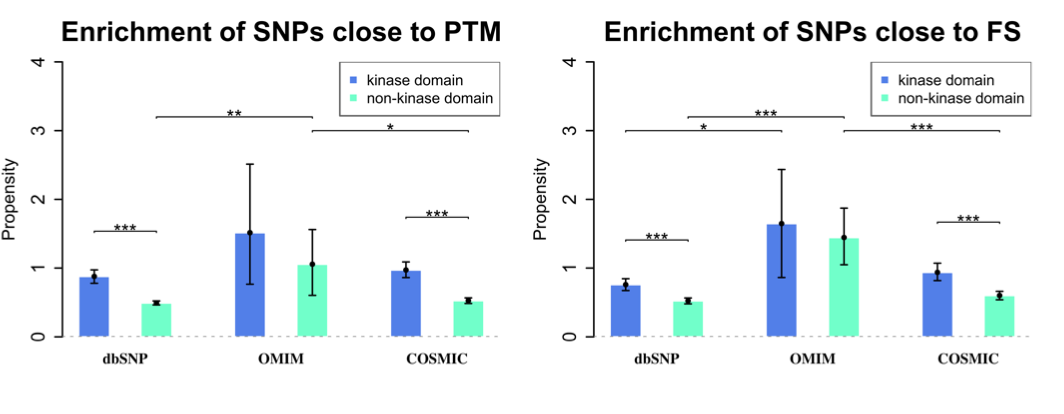
Figure S4. The enrichment of SNPs/variants close to PTMs and functional sites (FSs) was computed (in 3D space with cutoff 8 Å). Protein domains were divided into two categories, kinase and non-kinase domains. Our results show that SNPs/variants within kinase domains are enriched in the vicinity of PTMs or FSs compared to non-kinase domains. COSMIC variants are significantly enriched (Wilcoxon test: *p<0.05; **p<0.01; ***p<0.001) at the regions close to PTMs and FSs.


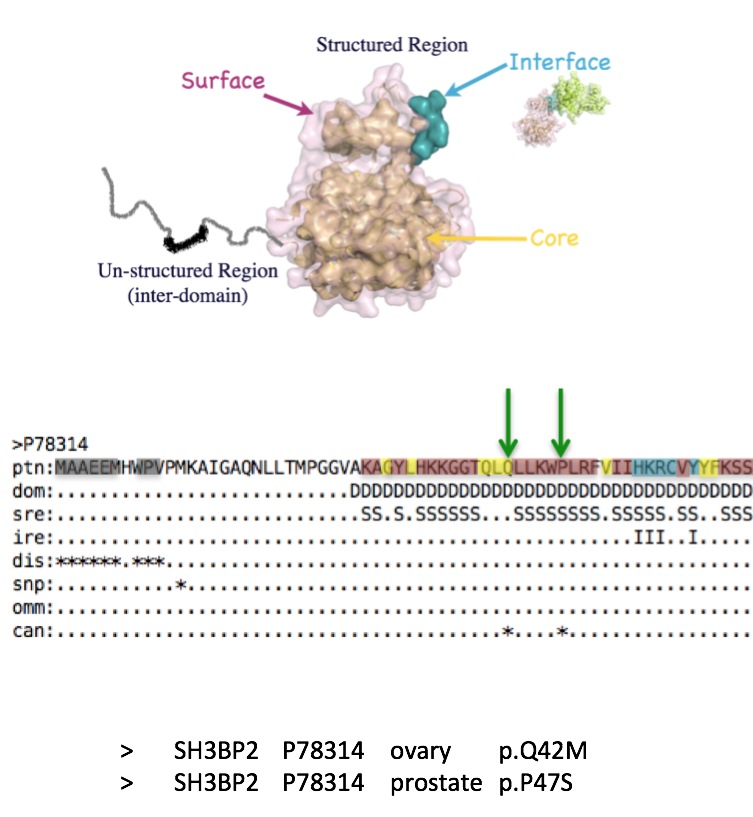


Figure S5. Overview of the structural regions and an exemplary sequence profile annotation of a protein. The sequence profile has been cropped for illustration purpose. Sequence lane annotations: *ptn*: protein sequence; *dom*: domain folded residues; *sre*: surface region residues; *ire*: interface region residues; *dis*: predicted disordered region residues; *snp*: SNP positions (common variants, other); *omm*: OMIM SNP positions; *can*: COSMIC somatic cancer variant positions. Green arrows point at two example cancer variant positions listed below the profile. Variant p.Q42M located in the core region (yellow residue), while variant p.P47S occurs in the surface region (pink residue).


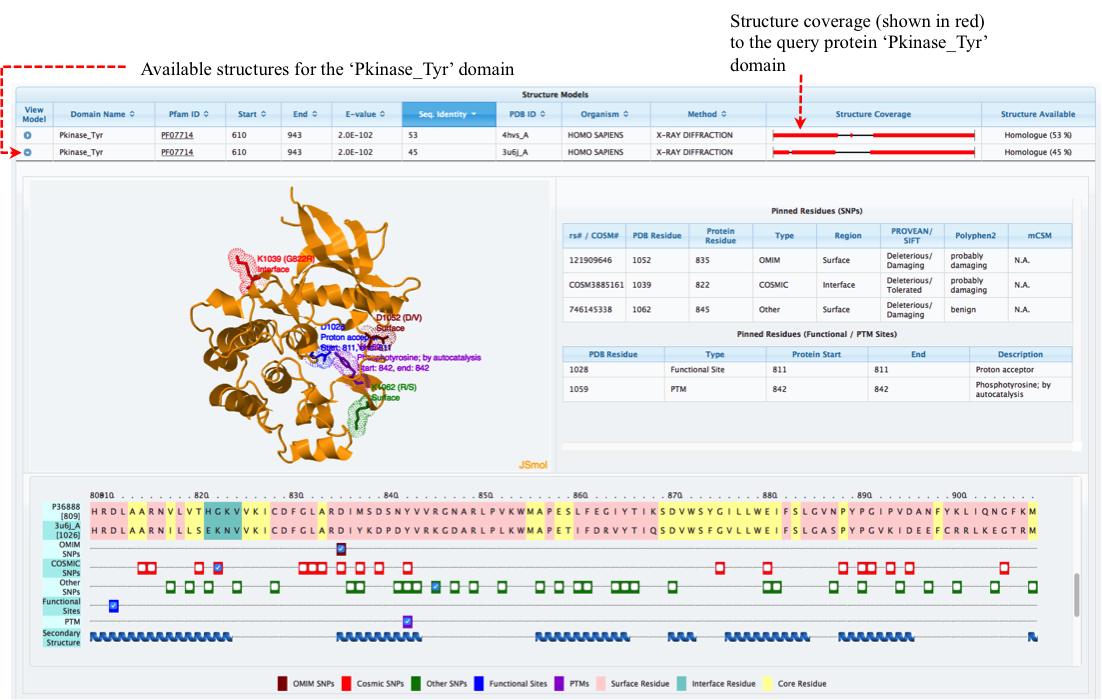


Figure S6. PinSnps analysis of the receptor-type tyrosine-protein kinase FLT3 (Uniprot AC P36888). Mapping of SNPs, functional sites and PTMs onto the rotatable protein structure was performed by selecting checkboxes in the opposite reference alignment, leading to the appearance of colour-coded annotation text and side-chain rendering in the structure viewer.


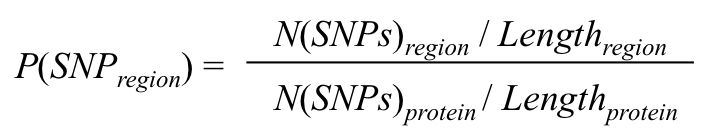


Equation S1. Formula for the analysis of regional enrichment/depletion of SNPs as propensity P(SNP_region_).


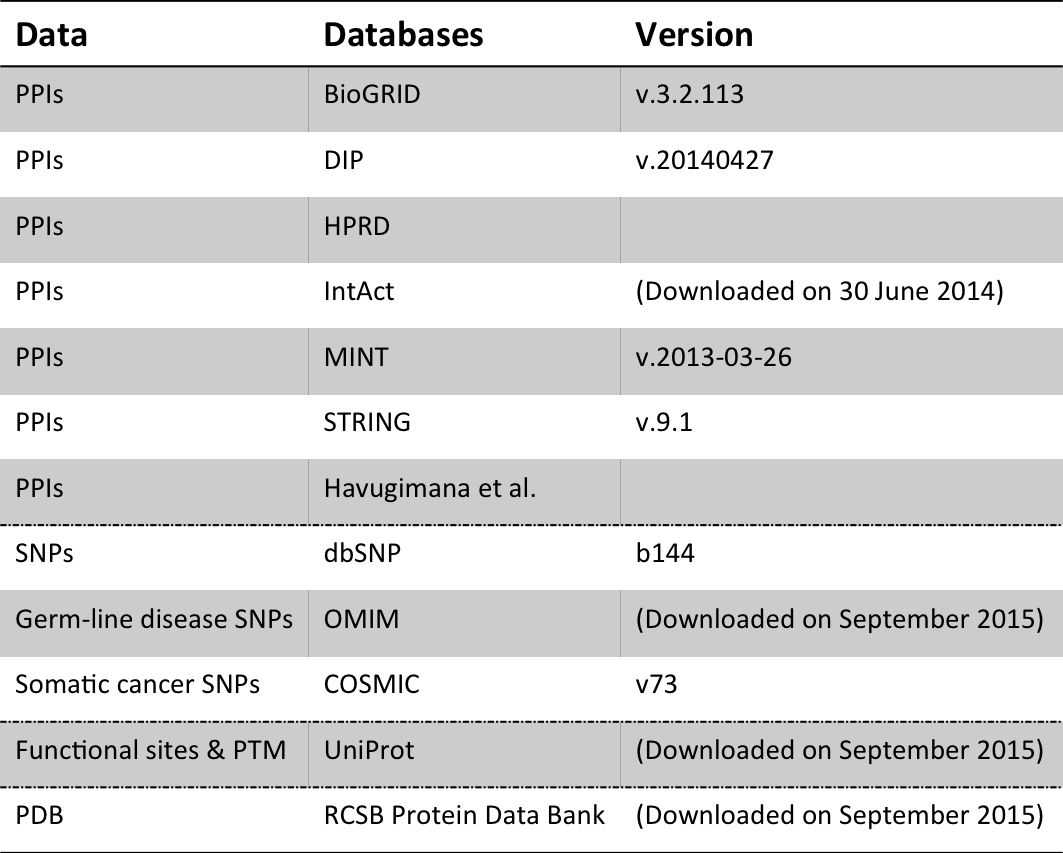


Table S1. Data sources.
